# Effects of titanium prepared platelet rich fibrin on facial nerve regeneration: an experimental study

**DOI:** 10.1016/j.bjorl.2020.11.014

**Published:** 2020-12-26

**Authors:** Fatma Şentürk, Osman Bahadır, Osman Aktaş, Ayşe Firuze Bıyık, Esra Ercan

**Affiliations:** a Bingöl Maternity and Children’s Hospital, Department of Otolaryngology, Bingöl, Turkey; b Karadeniz Technical University, Faculty of Medicine, Department of Otolaryngology, Trabzon, Turkey; c Karadeniz Technical University, Faculty of Medicine, Department of Physiology, Trabzon, Turkey; d Karadeniz Technical University, Faculty of Medicine, Department of Histology and Embryology, Trabzon, Turkey; e Çanakkale Onsekiz Mart University, Faculty of Dentistry, Department of Periodontology, Çanakkale, Turkey

**Keywords:** Facial nerve injury, Surgical treatment, Platelet-rich fibrin

## Abstract

**Introduction:**

Facial nerve damage is a condition that causes functional, psychological, and cosmetic problems; and treatment methods need to be improved.

**Objective:**

We investigated the efficacy of titanium-prepared platelet-rich fibrin as a healing enhancer at the region of transection of the facial nerve.

**Methods:**

Twenty-seven New Zealand male rabbits were used in this study, divided into three experimental groups. Group 1, the sham group (n = 7); Group 2, the suture group (n = 10); and Group 3, the suture + T-PRF group (n = 10). In Group 1, the facial nerve trunk was dissected, and no additional surgical intervention was performed. For Group 2, a transection was made to the facial nerve trunk and the nerve endings were sutured together. In Group 3, nerve endings were sutured after transection, and a titanium-prepared platelet-rich fibrin membrane was wrapped in a tube around the damaged area. All animals were followed up weekly for the presence of corneal reflex, whisker movement and low ears. Bilateral facial electromyography was performed both preoperatively and postoperatively at the 1st, 3rd, 5th, 7th, 10th weeks. Tissue samples obtained at the 10th week were histopathologically examined, and intra-group and inter-group comparisons were performed.

**Results:**

Subjects in Group showed improvement in whisker movement and ear drop one week earlier than Group 2. In Group 3, the nerve stimulation threshold required to trigger the compound muscle action potential had returned to values similar to the preoperative control values (11.31 ± 2.16 V) by 5 weeks postoperatively (12.51 ± 3.97 V), (*p* = 0.249).

**Conclusion:**

Titanium-prepared platelet-rich fibrin administration contributed to partial nerve healing both on a functional and an electrophysiological level.

## Introduction

The facial nerve is an important peripheral nerve due to its role in various functions. Its damage can cause functional, aesthetic, and psychological problems in individuals. In peripheral nerve repair, there has been no significant development in surgical treatment in recent years, and the functional results of nerve repair are still not perfect.^1^ Therefore, alternative treatments that increase nerve healing are needed in addition to surgical treatment.^2^

In recent years, the use of platelet-rich products has become widespread in the fields of medicine and dentistry. Platelet-rich fibrin (PRF) was originally developed by Choukroun in 2001, drawing attention to the potential of platelet-rich products.^3^ Growth factors and cytokines resulting from activation of platelets are trapped in the PRF network during centrifugation. During wound healing, these factors are released slowly over time,^4^, ^5^ accelerating bone and wound healing and improving the quality of healing.^6^ However, studies showing the effects of PRF on nerve regeneration are limited. Moreover, some argue that leukocytes are protected in the PRF membrane, and these leukocytes are effective in preventing infections and contributing to the regulation of the immune system.^7^

PRF has been modified by O’Connell, who used titanium tubes with high biocompatibility to avoid silica-related problems during blood collection.^8^ Although the PRF obtained with titanium tube is similar to the classic PRF described by Tunalı et al., the fibrin network structure is thicker and more highly organized.^9^

This study aims to evaluate in a rabbit model the functional, electrophysiological, and histopathological effects of titanium-prepared platelet-rich fibrin (T-PRF) that includes various growth factors on the regeneration of the facial nerve.

## Methods

### Surgical procedure

The research was carried out at Surgery Research and Application Center with the approval of the Animal Experiments Local Ethics Committee with the protocol number 2017/23. A total of 27 male New Zealand rabbits with normal bilateral facial nerves, each weighing 2000–3000 g, were kept under standard conditions (21–22 °C, 12 h light/dark cycle) without restrictions from feed or water. The experimental animals were randomly divided into three groups: Group 1, the sham group (n = 7); Group 2, the suture group (n = 10); and Group 3, the suture + T-PRF group (n = 10).

Prophylactically, 20–40 mg/kg cefazolin sodium was administered intramuscularly to subjects 1 h before and 1 h after the procedure. General anesthesia was provided via 35 mg/kg ketamine hydrochloride and 5 mg/kg xylazine hydrochloride administered intramuscularly.

The experimental procedure was carried out on the right facial nerve of each subject. After cleaning of the surgical area with a 10% povidone-iodine solution, a right preauricular approximately 5 cm incision was made through the skin and subcutaneous tissues. The masseteric part of the sphincter colli superficialis muscle was crossed, and the buccal branch of the facial nerve was made visible above the masseter muscle. The buccal branch was followed toward the proximal part of the facial nerve. The nerve truncus was revealed between the point of exit of the stylomastoid foramen and the bifurcation region.

**Figure 1:**
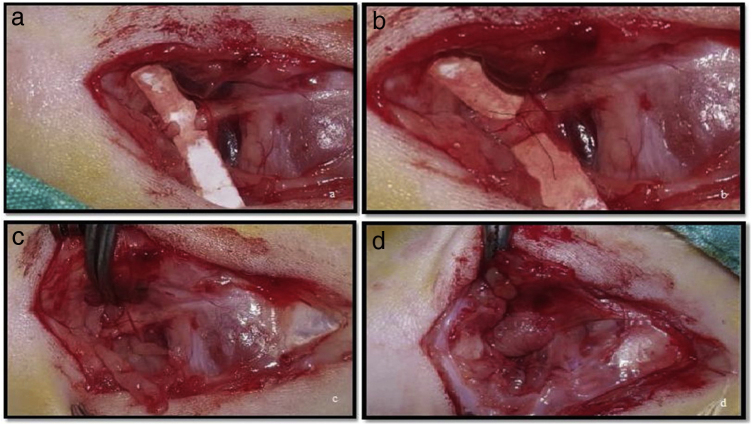
(a) Making a full-thickness incision to the facial nerve truncus; (b) the suturing of nerve endings; (c) the fitting of the PRF membrane; (d) wrapping of the PRF membrane around the facial nerve truncus.

After the exposure and dissection of the nerve in Group 1, the skin was sutured with 4.0 silk (ipek/silk USP 4/0, Doğpa Tıbbi Malzeme Tic. A.Ş., Turkey) without any additional treatment. In Group 2, a transection of the nerve trunk was made using a surgical microscope and microsurgical instruments (Fig. 1a). The nerve trunk was then repaired with 9.0 microsuture (Daylon® USP 9/0, Doğpa Tıbbi Malzeme Tic. A.Ş., Turkey), and two epineural sutures were made at 0° and 180° (Fig. 1b). In Group 3, following the transection and nerve suturing with 9.0 microsuture as performed in Group 2, each subject’s own T-PRF was wrapped around the nerve in a tube shape above the sutured area (Fig. 1c–d).

### Preparation of T-PRF

For each subject in Group 3, 10 mL of blood was removed from the right or left marginal ear artery. The drawn blood was rapidly transferred to two titanium 5 mL tubes and centrifuged at 3500 rpm for 15 min at room temperature using a table centrifuge (NF 400, Nuve, Turkey).^10^ After centrifugation, the clot (T-PRF) obtained was removed from the titanium tubes using a sterile tweezer. The resulting clot was separated from the red blood cells, crushed between gauze and formed into a membrane.

### Functional evaluation

After surgery, the whisker movement, ear drop, and corneal reflex of each subject were recorded weekly by the same observer. Evaluation of the corneal reflex used the 5-point rating scale identified by Borin et al.:^11^ 0, No visible closing movement; 1, Less than 1/3 narrowing in the palpebral fissure; 2, Narrowing in the palpebral fissure between 1/3 and 2/3; 3, More than 2/3 narrowing in the palpebral fissure; 4, Closure of the eye completely by touching the cornea.

Whisker movement and ear drop were evaluated weekly as either present or not.

### Electrophysiological evaluation

A total of six bilateral facial lectromyography (EMG) tests were performed, before surgery and at the 1st, 3rd, 5th, 7th, and 10th weeks after surgery by the same person. Anesthesia was applied to all subjects during the procedure. A stimulus was given using a BSLSTM stimulator module (BIOPAC® Systems Inc., USA) on the facial nerve truncus line below the stylomastoid foramen with a two-probe electrode. The supramaximal warning threshold level was determined by increasing the warning intensity in 1-V units. Samples were taken using the three-point needle electrodes from mimic muscles in the whiskers area. Biopack Student Lab Pro 3.7.7. software and Biopack MP36 hardware (BIOPAC® Systems Inc., USA) were used for registration.

The amount of stimulus given to determine the level of facial nerve warning threshold, compound muscle action potential (CMAP) maximum amplitude values, and nerve conduction velocity were measured.

### Histopathological evaluation

Each subject’s right facial nerve truncus was dissected from the surrounding tissues under anesthesia for histopathological evaluation. The truncus was removed from 5 mm proximal and 5 mm distal to the incision region. The left facial nerve truncus (i.e., one not operated on surgically) was exposed and a 1 cm part was excised. Then, euthanasia of all subjects was performed by exsanguination via right or left carotid artery incision.

Tissues were fixed in 10% formaldehyde within 48 h for light microscopic examination. Following fixation, they were washed in running tap water for 24 h. After being passed through alcohol solutions of increasing grade (70%, 90%, 96%, 100%) for dehydration, they were cleared with xylene solvent and embedded in paraffin blocks. With the help of a fully automated microtome (LEICA RM 2255, Tokyo, Japan), the paraffin blocks were trimmed in 10 μm sections until the tissue was reached, and 5 μm sections were taken from the tissue. Hematoxylin–Eosin staining was used to observe the general histological structure.

Vascular congestion, edema, vacuolization, collagen accumulation, myelin and axon degeneration axonal continuity, inflammatory cell, fibrosis, and Schwann cell proliferation were evaluated semi-quantitatively (for axonal continuity 1: continuous, 2: moderate, 3: weak; for Schwann cell proliferation 0: none, 1: severe, 2: moderate, 3: mild; for other parameters 0: none, 1: mild, 2: moderate, 3: severe). A light microscope (Olympus® BX 51, Japan) with attached digital camera (Olympus® DP 71, Japan) was used for taking photographs and measuring at 40× magnification. Myelin sheath thickness was measured using the Olympus Database Manual cellSens Life Science Imaging Software program, Version 510_UMA _Database_cellSens19-Krishna-en-00.

### Statistical analysis

All data were analyzed using the SPSS 22.0 statistics program. The Wilcoxon test and Kruskal–Wallis variance analysis were used in the histopathological evaluation. Post-hoc analysis was made using the Mann–Whitney *U* test, and p-values ​​obtained in post-hoc analysis were interpreted by applying the Bonferroni correction. Student’s *t*-test, bidirectional variance analysis, and repeated measures of variance analysis were used in the physiological evaluation. Dunnett’s test was used for the post-hoc calculation of bidirectional variance analysis; *p* < 0.05 was considered statistically significant.

## Results

Four of the subjects (two from each of Groups 1 and 2) died during the procedures. No other complications were observed. The study results of those subjects were included in the analysis until the weeks of their death.

### Functional findings

In Group 1, corneal reflexes did not differ from before to after surgery. In the weeks following surgery in Groups 2 and 3, an improvement was observed in the corneal reflex. In weekly followups, there was no statistically significant difference in corneal reflex between these two groups.

In Group 2, there was a drop in the right ear for all subjects in the first three weeks. Ear drop began to subside at week 4 and had improved in all subjects at week 7. In Group 3, the right ear drop started to heal in the 3rd week and had completely improved by the 5th week.

In Group 2, noticeable whisker movement was recorded from the 9th week. On the other hand, this was observed from the 8th week in Group 3. By the 10th week, whisker movement existed in seven out of eight subjects in Groups 2 and 8 out of ten subjects in Group 3.

### Electrophysiologic findings

Right side warning thresholds were significantly higher in Groups 2 and 3 compared to Group 1 until the 10th week. These values did not differ significantly between Groups 2 and 3 except in the 3rd postoperative week (Table 1).

**Table 1:** The right side warning threshold values ​​of the experimental groups (mean ± SD, volt).

	Group 1		Group 2		Group 3	
	Right	Left	Right	Left	Right	Left
Preoperative	10.64 ± 3.47	12.02 ± 5.13	8.64 ± 2.04	10.21 ± 4.39	11.31 ± 2.16	10.85 ± 3.96
1st week	10.21 ± 3.34	10.46 ± 2.46	19.32 ± 7.02	9.07 ± 3.70	23.72 ± 4.99	9.61 ± 2.10
3rd week	10.09 ± 3.58	8.57 ± 1.93	18.34 ± 4.76	6.94 ± 3.08	26.57 ± 9.92	7.52 ± 3.07
5th week	8.53 ± 1.50	8.20 ± 3.01	17.68 ± 6.45	8.56 ± 2.95	16.59 ± 4.19	8.24 ± 2.36
7th week	8.74 ± 2.52	8.67 ± 4.03	15.36 ± 4.14	8.53 ± 2.82	15.75 ± 3.06	7.55 ± 2.37
10th week	7.82 ± 3.25	8.86 ± 3.43	11.43 ± 2.88	7.21 ± 2.80	12.51 ± 3.97	7.54 ± 3.73

**Figure 2:**
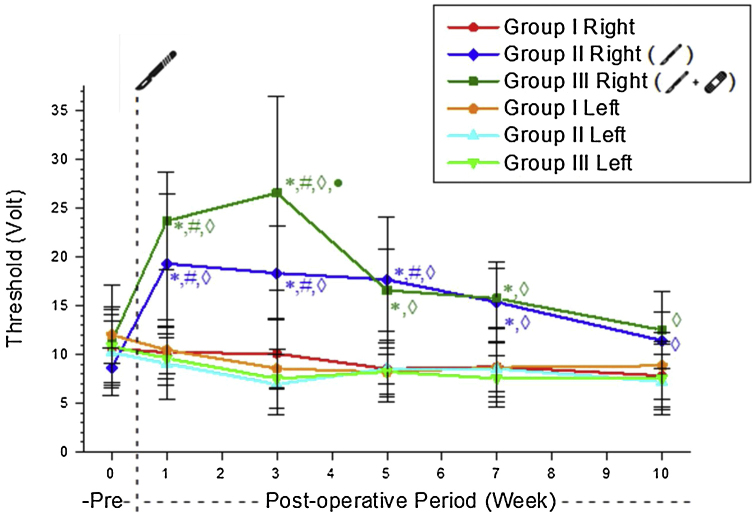
The warning threshold mean values ​​of the experimental groups. * Significantly different compared to Group 1 (*p* < 0.05). # Significantly different compared to the preoperative week (*p* < 0.05). ◊ Significantly different compared to the left side (*p* <  0.05); • Significantly different compared to Group 2 (*p* < 0.05).

Following the incision on the right side of Group 2, the warning threshold increased substantially. The increased threshold value after the incision was not different from the control values ​​in the postoperative 7th week in this group. In Group 3, the increased threshold value after the incision was not different from the control values ​​in the postoperative 5th week. The warning threshold of Group 3 recovered two weeks earlier than in Group 2 (Table 2, Fig. 2).

**Table 2:** Comparison of the right side postoperative warning threshold values with preoperative values within the groups (*p*-value).

	Preop-postop 1st week	Preop-postop 3rd week	Preop-postop 5th week	Preop-postop 7th week	Preop-postop 10th week
Group 1	1.000	0.999	0.780	0.865	0.609
Group 2	<0.001^a^	0,001^a^	0.004^a^	0.051	0.839
Group 3	<0.001^a^	<0.001^a^	0.249	0.438	0.996

a Significant differences *p* < 0.05.

**Figure 3:**
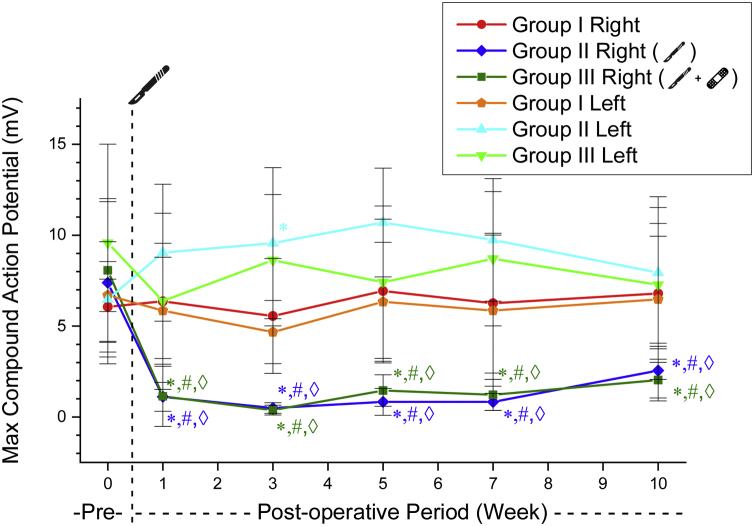
CMAP maximum amplitude values ​​in experimental groups. * Significantly different compared to Group 1 (*p* < 0.05). # Significantly different compared to the preoperative week (*p* < 0.05). ◊ Significantly different compared to the left side (*p* < 0.05).

The maximum amplitude values ​​of CMAP obtained on the right side decreased after surgery in Groups 2 and 3. These values ​​were significantly lower than in the sham group and their preoperative control values ​​in all weeks (*p* < 0.05). Groups 2 and 3 right-sided CMAP maximum amplitude values ​​did not differ significantly in the postoperative weekly follow-up (Table 3, Fig. 3).

**Table 3:** CMAP maximum amplitude values of the groups (mean ± SD) (millivolt).

	Group 1		Group 2		Group 3	
	Right	Left	Right	Left	Right	Left
Preoperative	6.06 ± 2.48	6.69 ± 0.89	7.39 ± 4.46	6.47 ± 3.16	8.06 ± 3.95	9.58 ± 5.42
1st week	6.36 ± 4.84	5.84 ± 2.94	1.11 ± 0.79	9.04 ± 3.76	1.14 ± 1.65	6.38 ± 3.17
3rd week	5.55 ± 3.16	4.67 ± 1.73	0.49 ± 0.29	9.56 ± 4.15	0.37 ± 0.27	8.62 ± 3.61
5th week	6.93 ± 3.95	6.33 ± 3.26	0.83 ± 0.73	10.70 ± 2.99	1.4 ± 0.86	7.41 ± 4.19
7th week	6.26 ± 3.84	5.85 ± 4.15	0.83 ± 0.46	9.74 ± 3.36	1.22 ± 0.85	8.70 ± 3.69
10th week	6.79 ± 4.73	6.47 ± 3.46	2.55 ± 1.50	7.93 ± 4.17	2.03 ± 1.14	7.27 ± 3.37

**Figure 4:**
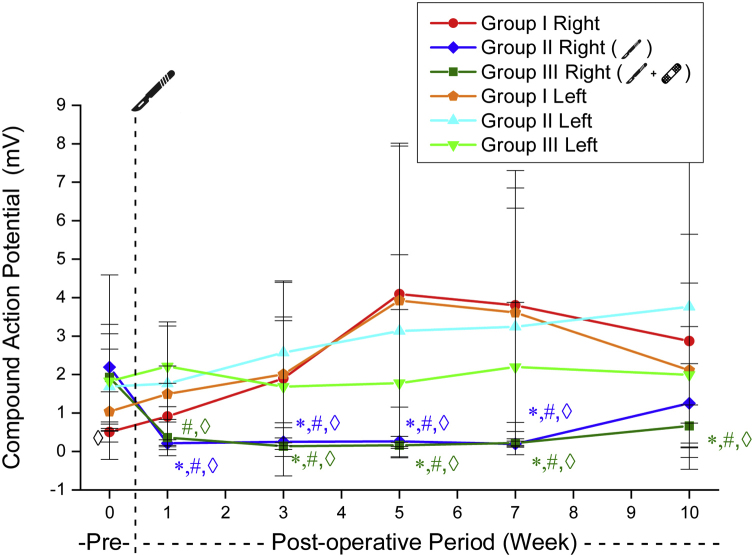
CMAP amplitude values ​​triggered by over-threshold stimulation. * Significantly different compared to Group 1 (*p* < 0.05). # Significantly different compared to the preoperative week (*p* < 0.05). ◊ Significantly different compared the left side (*p* < 0.05).

Similarly, the right side CMAP amplitude values obtained by suprathreshold stimulation decreased after surgery in Groups 2 and 3. In Group 2, this value was not significantly different from the preoperative values ​​at week 10 (*p* = 0.478). In group 3, this significant decrease from preoperative status persisted throughout all weeks (*p* < 0.05). These values ​did not differ significantly in postoperative weekly follow-ups in Groups 2 and 3 (Table 4, Fig. 4).

**Table 4:** CMAP amplitude values ​​triggered by over-threshold stimulation (mean ± SD) (millivolt).

	Group 1		Group 2		Group 3	
	Right	Left	Right	Left	Right	Left
Preoperative	0.50 ± 0.27	1.04 ± 0.51	2.19 ± 2.39	1.68 ± 0.97	1.92 ± 1.38	1.82 ± 1.23
1st week	0.91 ± 0.86	1.49 ± 0.72	0.21 ± 0.09	1.76 ± 1.60	0.36 ± 0.47	2.21 ± 1.04
3rd week	1.90 ± 2.53	2.00 ± 1.39	0.25 ± 0.10	2.57 ± 1.82	0.13 ± 0.08	1.68 ± 1.81
5th week	4.09 ± 3.85	3.92 ± 4.08	0.26 ± 0.13	3.13 ± 1.98	0.16 ± 0.77	1.77 ± 1.91
7th week	3.80 ± 3.04	3.61 ± 3.69	0.19 ± 0.07	3.24 ± 3.08	0.22 ± 0.10	2.19 ± 1.67
10th week	2.87 ± 2.77	2.11 ± 2.26	1.25 ± 1.03	3.76 ± 4.22	0.66 ± 0.54	1.99 ± 1.25

**Figure 5:**
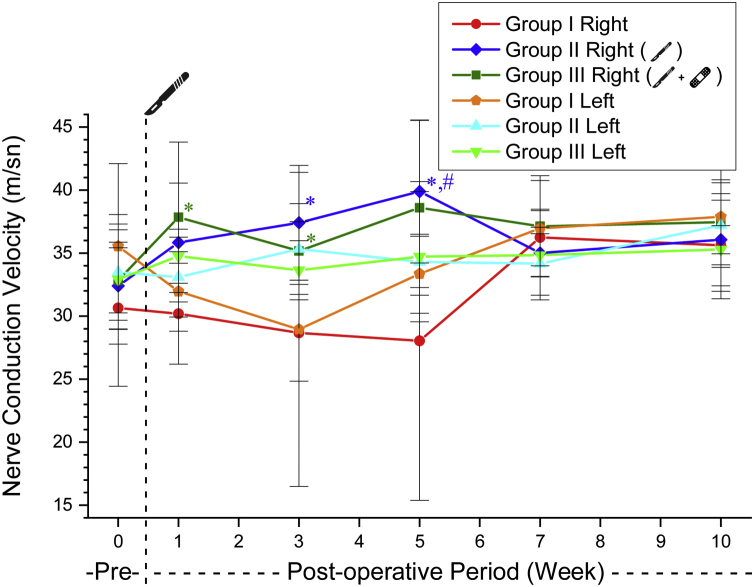
The nerve conduction velosity in the experimental groups. * Significantly different compared to Group 1 (*p* < 0.05). # Significantly different compared to the preoperative week (*p* < 0.05).

No significant difference was observed between Groups 2 and 3 in nerve conduction velocity values ​​before and after surgery (Table 5, Fig. 5).

**Table 5:** Nerve conduction velocity values of the groups (mean ± SD) (m/s).

	Group 1		Group 2		Group 3	
	Right	Left	Right	Left	Right	Left
Preoperative	30.64 ± 6.20	35.54 ± 6.55	32.40 ± 3.46	33.49 ± 3.81	32.84 ± 2.57	32.91 ± 5.14
1st week	30.19 ± 4.00	31.96 ± 3.15	35.83 ± 4.71	33.09 ± 3.19	37.84 ± 5.96	34.75 ± 2.14
3rd week	28.67 ± 3.83	28.94 ± 12.45	37.41 ± 4.54	35.32 ± 3.60	35.17 ± 2.32	33.63 ± 2.34
5th week	28.03 ± 12.64	33.35 ± 3.12	39.88 ± 5.67	34.29 ± 2.03	38.59 ± 6.93	34.71 ± 5.16
7th week	36.23 ± 2.24	36.95 ± 3.80	35.01 ± 3.35	34.16 ± 2.87	37.12 ± 4.01	34.83 ± 1.69
10th week	35.62 ± 1.55	37.88 ± 2.93	36.06 ± 3.65	37.21 ± 3.35	37.45 ± 5.48	35.28 ± 3.90

### Histopathological findings

Vacuolization and edema were observed semi-quantitatively less in Group 3 compared to Group 2, but this difference was not statistically significant. In Groups 2 and 3, the findings of vascular congestion, collagen infiltration, inflammatory cell, vacuolization, edema, fibrosis, Schwann cell proliferation, myelin degeneration, and axon degeneration were significantly higher than Group 1; axonal continuity and myelin sheath thickness were pointedly lower (Table 6). There was no significant difference between the right sides of Groups 2 and 3 in these parameters.

**Table 6:** Myelin sheath thickness of the groups (mean ± SD).

	Group 1 (n = 5)		Group 2 (n = 8)		Group 3 (n = 10)	
	Right	Left	Right	Left	Right	Left
Myelin sheath thickness (μm)	2.88 ± 0.31	3040 ± 0.48	1.08 ± 0.29	2.61 ± 0.32	1.03 ± 0.27	2.50 ± 0.28
Posthoc analysis of right-sided myelin sheath thickness findings between groups						*p*-Value
Group 1–Group 2						0.035^a^
Group 1–Group 3						0.002^a^
Group 2–Group 3						0.983

a Significant differences *p* < 0.05.

## Discussion

Facial nerve injury may develop as a consequence of trauma or surgery.^12^ In the case of nerve damage, the prognosis is quite poor without the help of surgical intervention and other biological agents, and the quality of life is significantly affected due to functional problems.^12^, ^13^

Platelet-rich products are classified in different ways according to their contents and methods of preparation; the first of these is platelet-rich plasma (PRP). This material, prepared by using tubes containing anticoagulants, is widely used in various fields due to its rich growth factors.^14^ Many studies have used this product as a healing agent in nerve damage.^15^, ^16^, ^17^

PRF, the second generation of platelet-rich products, is frequently preferred because unlike PRP it does not require anticoagulants and is easy to prepare and use. At least 8–10 mL of blood is needed to obtain a high-quality autogenous PRF membrane. The main reason rabbit models were selected for this study was for ease in obtaining a sufficient amount of blood to prepare the appropriate T-PRF material. In addition, PRF was preferred because the membrane obtained has a strong structure and can be applied locally in the form of a tube.

The literature contains various clinical and experimental studies carried out with T-PRF. In the study of Uzun et al., T-PRF was shown to be an effective and safe treatment method in multiple gingival defects.^18^ In a study comparing the uses of classical PRF and T-PRF, these two materials were reported to have positive clinical and radiological effects on recovery from intra-bone defects.^19^ It has also been reported that T-PRF increases epithelialization and reduces bleeding in mucosal wound healing in the palate.^20^

Although there are many studies to show that PRF provides positive contributions to bone and wound healing, a limited number of studies have evaluated its effect on nerve healing. Our study documents for the first time in the literature that T-PRF demonstrated nerve regeneration in the facial nerve. This finding will be beneficial for patients in the clinic, as an inexpensive and easily accessible means to reduce complications after trauma and surgeries, potentially providing a better recovery process.

Roth et al., in their studies evaluating the efficacy of autologous nerve graft versus PRF-coated vein graft, observed no significant difference between the two groups in functional scores in a model of sciatic nerve incision injury in rats.^21^ Lichtenfels et al. found a relationship with improved healing results in autologous nerve grafts in both PRP and PRF groups in a sciatic nerve incision injury model in rats.^22^ Torul et al. showed that the functional scores were similar in the PRF and PRGF groups in a model of sciatic nerve crush-type damage in rats.^23^ Şenses et al. reported that local PRF application showed no positive effect on functional evaluation in a sciatic nerve incision injury model in rats.^1^ In this study, we showed that the application of T-PRF in addition to sutures accelerates healing in terms of the whisker movement and ear drop.

Electrophysiological tests allowed evaluation of axonal regeneration at different stages of healing, and are one of the best methods used to evaluate peripheral nerve healing.^24^ The prolongation of latency is the increase of the required minimum stimulation intensity to trigger an action potential in the muscle. In other words, the increase of the stimulus threshold and the decrease of the amplitude voltage of the response to the stimulus of the same intensity reflect the deterioration of the neuromuscular function. After damage to the nerve, it is expected that the latency will increase, the warning threshold will increase, and the amplitude will decrease. These values are​ impaired with nerve damage, and the approach of these values ​​toward the control values ​​with recovery time are signs of improvement. In this study, repetitious EMGs were performed after surgery with the aim of demonstrating the course of recovery electrophysiologically.

In their electrophysiological measurements, Torul et al. showed that the amplitude was better in the PRF group and that PRF did not contribute to the latency parameter.^23^ In the study of Bayram et al., the positive effect of local PRF application on amplitude and conduction velocity could not be demonstrated ​​in the crush-type damage model in the rabbit sciatic nerve.^25^ Similarly, Şenses et al. remarked that PRF did not contribute to nerve conduction velocity and distal latency.^1^ In this study it was observed that local PRF application did not contribute to nerve conduction velocity, as in the Bayram and Senses studies. Additionally, the nerve stimulation threshold value required to trigger CMAP became similar to that of the preoperative control values in the local PRF application ​​at the postoperative 5th week, a 2-week improvement. This situation shows that T-PRF contributes to earlier recovery in the required warning threshold value to trigger CMAP. In a study on rats, it has been shown that axon diameter and myelin sheath thickness increase up to 50 weeks after sciatic nerve repair.^26^ Contrary to this, Farrag et al. expressed that an 8 week recovery period was sufficient to observe successful results on the facial nerve incision model in their study on rats.^15^ In the study of Lichtenfels et al., PRF application did not contribute to axon diameter, myelin thickness, or nerve fiber density.^22^ Roth et al. reported that the use of the PRF-coated vein graft and the autologous nerve graft had similar histopathological results,^21^ while Torul et al. showed that PRF did not contribute to myelin sheath thickness.^23^ Bayram et al. observed remyelination, creation of vascular formation, large-scale axon damage, and creation of collagen formation in histological evaluation, and reported that these findings were more apparent in their PRF group.^25^ Şenses et al. reported that the use of PRF membrane after nerve incision did not contribute to healing histologically.^1^ In the present study, it was determined that the use of T-PRF in addition to sutures did not contribute to histological healing or myelin sheath thickness. This result can be explained by the hypothesis that the T-PRF membrane does not prevent collagen infiltration despite stimulating wound healing in the region, causes inflammation due to the leukocytes it contains, and increases the time required for the completion of myelization and axonal regeneration.

## Conclusion

In this study, local T-PRF membrane application positively contributes to observable improvement in facial movements following healing after facial nerve injury. Electrophysiologically, the warning threshold findings were determined to recover earlier. Longer followup studies with standard methods, in which surgical and treatment protocols can be refined, can be of benefit in clarifying the effects of PRF on nerve healing.

## Funding

This research was supported by 10.13039/501100004045Karadeniz Technical University Scientific Research Project Coordination Unit (Project code: TTU-2018-6999).

## Conflicts of interest

The authors declare no conflicts of interest.
